# Supplementary material for: Consensus Sequences for Gag and Pol Introduced into HIV-1 Clade B Laboratory Strains Differentially Influence the Impact of Point Mutations Associated with Immune Escape and with Drug Resistance on Viral Replicative Capacity
Source: Viruses. 2025 Jun 12;17(6):842. doi: 10.3390/v17060842 (PMC12197770; doi:10.3390/v17060842)
Supplement: Supplementary file 1 [file viruses-17-00842-s001.zip › viruses-3473789-supplementary.pdf]

Supplementary Materials:

Supplementary Table S1: Bold faced nucleotides represent changes relative to pNL4-3, the respective triplets are underlined. Changes derived from a prior mutation are marked by underlined triplets as well.

Oligonucleotide primers (5' à 3')	Amino acid sequence
**Consensus variant primers:**	
CONS gag_1 F: $K_{15}R$:	AGCGGGGGAGAATTAGAT **A**GA TGGGAAAAAATT
CONS gag_1 R: $K_{15}R$:	ATCTAATTCTCCCCCGCTTAATACCGAC
CONS gag_2 F: $Q_{28}K$:	GCCAGGGGGAAAGAAA **A**AA TATAAACTAAAA
CONS gag_2 R: $Q_{28}K$:	TTTCTTTCCCCCTGGCCTTAACCGAA
CONS gag_3 F: $I_{82}V$:	ATCATTATATAATACA **G**TA GCA **AC**C CTCTATTGTG
CONS gag_3 R: $I_{82}V$:	TGTATTATATAATGATCTAAGTTCTT
CONS gag_4 F: $V_{84}T$:	TTATATAATACAGTAGCA **ACC** CTCTATTGTGTGC
CONS gag_4 R: $V_{84}T$:	TGCTACTGTATTATATAATGATCTAAGTTC
CONS gag_5 F: $D_{93}E$:	TGTGTGCATCAAAGGATA GA**A** GTAAAAGACAC
CONS gag_5 R: $D_{93}E$:	TATCCTTTGATGCACACACACAATAGAGG
CONS gag_6 F: $D_{102}E$:	GACACCAAGGAAGCCTTA GA**A** AAGATAGAGGAA
CONS gag_6 R: $D_{102}E$:	TAAGGCTTCCTTGGTGTCTTTTACTTCTA
CONS gag_7 F: $N_{125}S$:	GCAGCTGACACAGGAAAC A**G**C AGCCAGGTCAGCCA
CONS gag_7 R: $N_{125}S$:	GTTTCCTGTGTCAGCTGCTGCTTGCTGTGC
CONS gag_8 F: $H_{252}N$:	CAAATAGGATGGATGACA **A**AT AATCCACCTATCCC
CONS gag_8 R: $H_{252}N$:	TGTCATCCATCCTATTTGTTCCTGAAGGGTA
CONS gag_9 F: $G_{341}A$:	TTAAAAGCATTGGGACCA G**C**A GCGACACTAGAAGAA
CONS gag_9 R: $G_{341}A$:	TGGTCCCAATGCTTTTAAAATAGTCTTACAAT
CONS gag_10 F: $P_{373}S$:	ATGAGCCAAGTAACAAAT **U**CA GCTACCATAATGATA
CONS gag_10 R: $P_{373}S$:	ATTTGTTACTTGGCTCATTGCTTCAGCCAAAA
CONS gag_11 F: $I_{378}M$:	AAATCCAGCTACCATAATG AT**G** CAGAAAGGCAATT
CONS gag_11 R: $I_{378}M$:	CATTATGGTAGCTGGATTTGTTACTTGGCT
CONS gag_12 F: $K_{380}R$:	GCTACCATAATGATGCAG **CG**A GGCAATTTTAGG
CONS gag_12 R: $K_{380}R$:	CTGCATCATTATGGTAGCTGGATTTGTTA
CONS gag_13 F: $S_{494}N$:	CCTCAGATCACTCTTTGGC A**A**C GACCCCTCGTCACA
CONS gag_13 R: $S_{494}N$:	GCCAAAGAGTGATCTGAGGGAAGCTAAAG
CONS pol_1 $Q_{257}K$ F:	TGCATGCCTGCAGGGTTAAAA **A**AG AAA AAA TCA
CONS pol_1 $Q_{257}K$ R:	TTTTAACCCTGCAGGCATGCAAGCTTGGCGTA
CONS pol_2 $C_{317}S$ F:	GATCACCAGCAATATTCCAG **A**GT AGCATGACA
CONS pol_2 $C_{317}S$ R:	CTGGAATATTGCTGGTGATCCTTTCCATCC
CONS pol_3 $R_{432}K$ F:	TTTATGCAGGGATTAAAGTA A**A**G CAATTATGTA
CONS pol_3 $R_{432}K$ R:	TACTTTAATCCCTGCATAAATCTGACTTGCC
CONS pol_4 $V_{448}I$ F:	CCAAAGCACTAACAGAAGTA **A**TA CCACTAACA
CONS pol_4 $V_{448}I$ R:	TACTTCTGTTAGTGCTTTGGTTCCCCTAAG
CONS pol_5 $K_{513}R$ F:	AAATATGCAAGAATG A**G**A GGTGCCCACA
CONS pol_5 $K_{513}R$ R:	CATTCTTGCATATTTTCCTGTTTTCAGATTTTT
CONS pol_6 $I_{590}V$ F:	AGTTAGAGAAAGAACCCATA **G**TA GGAGCAGAA
CONS pol_6 $I_{590}V$ R:	TATGGGTTCTTTCTCTAACTGGTACCATAA
CONS pol_7 $P_{623}S$ F:	GAGGAAGACAAAAAGTTGTC **T**CC CTAACGGAC
CONS pol_7 $P_{623}S$ R:	GACAACTTTTTGTCTTCCTCTGTCAGTTAC
CONS pol_8 $V_{787}I$ F:	GTACACATTTAGAAGGAAAA **A**TT ATCTTGGTAGCAGTTC
CONS pol_8 $V_{787}I$ R:	TTTTCCTTCTAAATGTGTACAATCTAGCTGCC
CONS pol_9 $V_{828}I$ F:	GAAGATGGCCAGTAAAAACA **A**TA CATACAGAC
CONS pol_9 $V_{828}I$ R:	TGTTTTTACTGGCCATCTTCCTGCTAATTT
CONS pol_10 $I_{866}V$ F:	ATCCCCAAAGTCAAGGAGTA **G**TA GAATCTATG
CONS pol_10 $I_{866}V$ R:	TACTCCTTGACTTTGGGGATTGTAGGGAAT

CONS pol_11 V$_{949}$L F:	ACAGGGACAGCAGAGATCCA **C**TT TGGAAAGGA
CONS pol: 11 V$_{949}$L R:	TGGATCTCTGCTGTCCCTGTAATAAACCCG
**KK10-associated escape mutation primers:**	
L$_{268}$M_F:	CTATAAAAGATGGATAATC **A**TG GGATTAAAT
L$_{268}$M_R:	GATTATCCATCTTTTATAGATTTCTCCTACT
R$_{264}$K_F:	CCAGTAGGAGAAATCTATAAA A**A**A TGGATAATCCTG
R$_{264}$K_R:	TTTATAGATTTCTCCTACTGGGATAGGTGG
S$_{173}$A_F:	CAGAAGTAATACCCATGTTT **G**CA GCATTATCAG
S$_{173}$A_R:	GATTATCCATTTTTTATAGATTTCTCCTACT
L$_{268}$MonR$_{264}$K_F:	TATAAA AAA TGGATAATC **A**TG GGATTAAATAAA
L$_{268}$MonR$_{264}$K_R:	GATTATCCATTTTTTATAGATTTCTCCTACT
**Drug resistance mutation primers:**	
N$_{155}$H_F:	GTAA TAGAATCTAT G **C**AT AAAGAA TTAA
N$_{155}$H_R:	CATAGATTCTATTACTCCTTGACTT
N$_{155}$H on CONS_F:	GTAGTA G AATCTATG **C**AT AAAGAATTAA
N$_{155}$H on CONS_R:	CATAGATTCTA C TACTCCTTGACTT
D$_{30}$N_F:	ATACAGGAGCAGAT **A**AT ACAGTATTAG,
D$_{30}$N_R:	ATCTGCTCCTGTATCTAATAGAG
L$_{76}$V_F:	GCTATAGGTACAGTA **G**TA GTAGGACCTA
L$_{76}$V_R:	TACTGTACCTATAGCTTTATGTCCG
I$_{84}$V_F:	TACACCTGTCAAC **G**TA ATTGGAAGA
I$_{84}$V_R:	GTTGACAGGTGTAGGTCCTACTAATAC
K$_{65}$R_F:	GTAT TTGCCATAAAG A**G**A AAAGACAGTA
K$_{65}$R_R:	CTTTATGGCAAATACTGGAGTATTG
K$_{101}$P_F:	CATCCTGCAGGGTTA **CC**A CAGAAAAAAT
K$_{101}$P_R:	TAACCCTGCAGGATGTGGTATTCCT
K$_{101}$P on CONS_F:	CATCCTGCAGGGTTA **CC**AAAG AAAAAAT
K$_{101}$P on CONS_R:	TAACCCTGCAGGATGTGGTATTCCT
L$_{100}$I_F:	CCACATCCTGCAGGG **A**TA A AACAGAAAA
L$_{100}$I_R:	CCCTGCAGGATGTGGTATTCCTAAT
L$_{100}$I on CONS_F:	CCACATCCTGCAGGG **A**TA AAA AAG AAAA
L$_{100}$I on CONS_R:	CCCTGCAGGATGTGGTATTCCTAAT
Q$_{151}$M_F:	AATGTGCTTCCA **A**TG GGATGG
Q$_{151}$M_R:	TGGAAGCACATTGTACTGATATCTAATC
CONS Pro-D$_{30}$N_F:	GCTCTATTAGATACAGGAGCAGAT **A**AT ACAGTATTAGAAGAAATGAATT
CONS Pro-D$_{30}$N_R:	AATTCATTTCTTCTAATACTGTA AT**T** TCTGCTCCTGTATCTAATAGAGC
CONS Pro-L$_{76}$V_F:	GCGGACATAAAGCTATAGGTACAGTA **G**TA GTAGGACCTACA
CONS Pro-L$_{76}$V_R:	TGTAGGTCC TA**C** TACTACTGTACCTATAGCTTTATGTCCGC
CONS M$_{184}$V_F:	CAGACATAGTCATCTATCAATAC **G**TG GATGATTTGTATGTAGGATCT
CONS M$_{184}$V_R:	AGATCCTACATACAAATCATC CA**C** GTATTGATAGATGACTATGTCTG
RT-L$_{100}$I_F:	GCTTGCATGCCTGCAGGG **A**TA AAACAGAAAAAATCAGT
RT-L$_{100}$I_R:	ACTGATTTTTTCTGTTT TA**T** CCCTGCAGGCATGCAAGC
RT-K$_{101}$P_F:	CCAAGCTTGCATGCCTGCAGGGTTA **CC**A CAGAAAAAATCAGT
RT-K$_{101}$P_R:	ACTGATTTTTTCTG T**GG** TAACCCTGCAGGCATGCAAGCTTGG
CONS RT-L$_{100}$I_F:	CAAGCTTGCATGCCTGCAGGG **A**TA AAAAAGAAAAAATCAGTAAC
CONS RT-L$_{100}$I_R:	GTTACTGATTTTTTCTTTTT TA**T** CCCTGCAGGCATGCAAGCTTG
CONS RT-K$_{101}$P_F:	AAGCTTGCATGCCTGCAGGGTTA **CC**AAAG AAAAAATCAGTAACAGTAC
CONS RT-K$_{101}$P_R:	GTACTGTTACTGATTTTTT CTTT**GG** TAACCCTGCAGGCATGCAAGCTT

Supplementary Table S2: Statistical analysis of the different viral clones with regard to the respective figures.

Supplementary Table S2

Statistical data Figure 2

Virus variant	mean value	95% CI	p-value against NL4-3
NL4-3	0.999	0.903 to 1.095	-
Cons gag/pol	0.402	0.306 to 0.498	p<0.001
Cons pol	0.458	0.362 to 0.554	p<0.001
Cons gag	0.534	0.437 to 0.630	p<0.001

Statistical data Figure 3

Virus variant	mean value	95% CI	p-value against Cons gag
Cons gag	1	0.818 to 1.180	-
RK on Cons gag	0.140	0 to 0.320	p<0.001
RKLM on Cons gag	0.288	0.109 to 0.468	p<0.001
SALM on Cons gag	1.000	0.820 to 1.180	not significant
SA on Cons gag	1.133	0.953 to 1.313	not significant
LM on Cons gag	1.219	1.039 to 1.401	not significant
SARK on Cons gag	1.363	1.184 to 1.543	not significant
SARKLM Cons gag	1.554	1.375 to 1.736	p<0.05

Statistical data Figure 4

	NL4-3		
Virus variant	mean value	95% CI	p-value against NL4-3
NL4-3	1	0,863 to 1,137	-
NL4-3 + D30N	0.894	0.757 to 1.031	not significant
NL4-3 + K65R	0.791	0.654 to 0.929	<0.05
NL4-3 + L76V	0.228	0.091 to 0.365	<0.01
NL4-3 + I84V	0.579	0.443 to 0.716	<0.01
NL4-3 + L100I	1.058	0.920 to 1.195	not significant
NL4-3 + K101P	0.844	0.707 to 0.981	<0.05
NL4-3 + Q151M	0.524	0.387 to 0.661	<0.01
NL4-3 + N155H	0.462	0.325 to 0.599	<0.01
NL4-3 + Y181C	0.911	0.774 to 1.048	not significant
NL4-3 + M184V	0.700	0.562 to 0.837	<0.01

Statistical data Figure 5

	NL4-3		
Virus variant	mean value	95% CI	p-value against Cons gag/pol
Cons gag/pol	0.503	0.366 to 0.641	-
Cons gag/pol + D30N	0.343	0.206 to 0.480	<0.05
Cons gag/pol + K65R	0.782	0.645 to 0.919	<0.01
Cons gag/pol + L76V	0.137	0.0005 to 0.274	<0.01
Cons gag/pol + I84V	0.317	0.180 to 0.454	<0.05
Cons gag/pol + L100I	0.748	0.610 to 0.885	<0.05
Cons gag/pol + K101P	0.486	0.348 to 0.623	not significant
Cons gag/pol + Q151M	0.351	0.214 to 0.488	<0.05
Cons gag/pol + N155H	0.278	0.141 to 0.416	<0.05
Cons gag/pol + Y181C	0.527	0.390 to 0.664	not significant
Cons gag/pol + M184V	0.711	0.573 to 0.848	<0.05

Supplementary Table S3: The individual infection rates of the virus variants are listed in relation to the respective viral backbone and compared with each other.

Supplementary Table S3		
backbone / mutation	NL4-3	Cons *gag/pol*
no mutation	1.00	1.00
D30N	0.89	0.69
K65R	0.79	1.37
L76V	0.23	0.27
I84V	0.58	0.57
L100I	1.06	1.34
K101P	0.84	0.97
Q151M	0.52	0.71
N155H	0.46	0.56
Y181C	0.91	1.05
M184V	0.70	1.40
